# A novel beam stopper-based approach for scatter correction in digital planar radiography

**DOI:** 10.1038/s41598-023-32764-5

**Published:** 2023-05-31

**Authors:** N. Sakaltras, A. Pena, C. Martinez, M. Desco, M. Abella

**Affiliations:** 1 Departamento de Bioingeniería, Universidad Carlos III de Madrid, Avda. de la Universidad 30, 28911 Leganés, Madrid, Spain; 2 Instituto de Investigación Sanitaria Gregorio Marañón, Madrid, Spain; 3 Centro Nacional Investigaciones Cardiovasculares (CNIC), Madrid, Spain; 4 Centro de Investigación en Red en Salud Mental (CIBERSAM), Madrid, Spain

**Keywords:** Biomedical engineering, Imaging techniques

## Abstract

X-ray scatter in planar radiography degrades the contrast resolution of the image, thus reducing its diagnostic utility. Antiscatter grids partially block scattered photons at the cost of increasing the dose delivered by two- to four-fold and posing geometrical restrictions that hinder their use for other acquisition settings, such as portable radiography. The few software-based approaches investigated for planar radiography mainly estimate the scatter map from a low-frequency version of the image. We present a novel method for scatter correction in planar imaging based on direct patient measurements. Samples from the shadowed regions of an additional partially obstructed projection acquired with a beam stopper placed between the X-ray source and the patient are used to estimate the scatter map. Evaluation with simulated and real data showed an increase in contrast resolution for both lung and spine and recovery of ground truth values superior to those of three recently proposed methods. Our method avoids the biases of post-processing methods and yields results similar to those for an antiscatter grid while removing geometrical restrictions at around half the radiation dose. It can be used in unconventional imaging techniques, such as portable radiography, where training datasets needed for deep-learning approaches would be very difficult to obtain.

## Introduction

X-ray scatter causes a loss of image contrast in planar radiography. The use of antiscatter grids enables the removal of a significant part of the scattered photons reaching the detector, although this comes at the cost of a two- to four-fold increase in the radiation dose delivered^1^. Furthermore, grids are optimized for a specific source-to-detector distance and position, thus hindering their use for other acquisition settings, such as portable radiography. The introduction of digital radiography has fostered the development of more modern scatter reduction techniques.

Scatter correction techniques can be divided into two categories: (1) those based on hardware; and (2) those based on image processing. One of the hardware-based strategies using a beam stopper placed between the X-ray source and the patient to extract the scatter signal from the shadow area. In^2,3^, the authors made use of small-area beam stoppers (lead strips, cubes, or cylinders) placed on the sample. A major drawback of these implementations is that the scatter signal for the whole volume is represented by a single local scatter estimation. Later studies used an extra acquisition with a larger beam stopper designs covering the whole detector area. In^4^, the authors substituted the two acquisitions by a single acquisition with two phosphor plates: the first, to acquire the total image, and the second, placed after a beam stopper, which comprises a polymethylmethacrylate (PMMA) plate with an embedded mesh of lead cylinders, to acquire the scatter signal. However, this strategy is incompatible with current digital detectors.

Alternatively, other methods used a mesh of holes as beam stopper and assume that the projection of these holes contains only primary signal. They estimate the scatter map from the subtraction of those hole regions from the image without the beam stopper. In^5^, a lead plate with a mesh of holes was used for digital fluoroscopy. Although the method showed a decreased signal-to-noise ratio (SNR) in the corrected images, it was evaluated using a very simple phantom made up of various aluminum disks in a homogeneous block of PMMA, with no ground truth for comparison. In^6^, the authors added an empirical global correction factor based on the ratio of the flat field images with and without the beam stopper to compensate for the lower scatter radiation when using the beam stopper. In^7^, the authors substituted the single global factor by a correction map based on the same ratio of flat field images. Nevertheless, these correction factors did not account for differences in probability of Compton scattering among different tissue types (bone, soft tissue, lung); this is especially important in chest radiography. In^8–10^, the authors used the Scatter to Primary Ratio (SPR), obtained by dividing the scatter map generated as in the previous methods by the beam stopper projection, which was interpolated to obtain a complete SPRc map. The corrected image was then obtained by multiplying the full field projection by the factor 1/(1 + SPRc). The main problem with these methods is that they assume that only primary signal reaches the holes region, without additional evidence or support from previous literature.

Regarding computed tomography (CT), in^11,12^, the authors inserted a thin lead plate with slits between the object and the detector to obtain a second acquisition for each projection angle. Subsequent works in CT obviated the need for two acquisitions by using iterative reconstruction algorithms to account for the data missing in the projections due to the beam stopper^13,14^. In^15^, the authors used a moving plate with a mesh of holes and interpolated the missing data before reconstruction, although this was only evaluated on simulations. The latter methods compensated for the missing data using the available tomographic information, which is not obtainable in radiography. A different type of blocker made of copper or aluminum with a checkerboard pattern ﻿was suggested for CT in^16–18^. This blocker does not stop the beam, but modulates the primary signal, which is richer in higher frequencies than the scatter signal, thus enabling their separation in the Fourier domain. Although this aproach needs a single projection with the modulator, the pattern is still visible in the projection hindering its use for radiography^19^.

Alternatively, image processing methods do not require any modification of the system hardware. In^20^, the authors created a database of precomputed scatter maps obtained by simplified simulations, considering only single scattering events of sequentially thicker PMMA plates (as a soft tissue equivalent). These precomputed maps were used to estimate the scatter map corresponding to each projection by considering the thickness of soft tissue traversed, which was obtained from a preliminary reconstructed CT volume. Image processing methods in planar X-ray are mostly based on the low pass nature of the scatter. In^3^, the authors tested various global convolution kernels, choosing the optimal one based on the scatter measurement behind a thin lead strip. Kotre et al.^21^ improved the scatter map by incorporating a preliminary step to remove the bone from the projections, although this required a weighting factor that had to be manually tuned. The main drawback of these works is that a single global convolution kernel cannot represent the scatter distribution of a chest radiograph. The authors in^22^ made use of different convolution kernels for the two major regions of a chest radiograph, namely, the lungs and mediastinum, which where manually chosen depending on the region of interest. Although they achieved better results than the single convolution kernel approach, the authors mentioned that tissue boundaries, i.e., lung-to-spine and spine-to-heart, required further investigation. Later, in^23–25^, the authors explored more complex kernels to better estimate variations in the thickness of the sample and the scatter in the interfaces of the tissues. However, these kernels were designed considering a homogeneous object comprising only soft tissue. Such an approach could prove problematic in clinical practice since bony structures generate and absorb scatter differently from soft tissue. In^26–30^, the authors evaluated the image dehazing method proposed by Meng et al.^31^ to obtain a scatter map in radiography based on the alternating direction method of multipliers (ADMM)^32^. However, simultaneous correction of lungs and spine in chest radiography was not possible because of the need for a specific parameter that had to be empirically tuned depending on the intensity of each region. Similar approaches were proposed for dual energy acquisitions, applying the ADMM algorithm prior to base material decomposition^30^.

More recently, deep-learning approaches have been proposed to estimate the scatter map in cone-beam CT. Maier et al.^33^ used a convolutional neural network based on U-Net to predict the scatter map obtained by a Monte-Carlo simulation, albeit in a shorter time. In a later work^34^, the authors proved that their method generalizes well to different tube voltages, noise, and various anatomical regions, provided they are appropriately represented in the training dataset. In^35^, Jiang et al. applied the residual learning framework to mitigate the problem of vanishing gradients and dropout and thus avoid overfitting. In^36^, the possibilities of transfer learning in the field were demonstrated using fine-tuned networks on full-fan scan data with half-fan scans. The main limitation of deep-learning approaches is the need for large datasets with and without scatter correction, while these pairs of acquisitions are rarely available in clinical practice. This is particularly hard when dealing with nonstandard geometries, such as those of portable radiography.

In this work, we present a novel method for scatter correction in planar radiography that avoids the high increase in dose delivered and the geometrical restrictions associated with the use of antiscatter grids. This paper extends our previous preliminary results reported in^37^. The method is based on a second acquisition with a perforated plate (beam stopper) placed between the source and the patient and in which scatter samples are found in the shadow of the plate. The main advantage of the method is that the correction is based directly on measurements of the scatter component over the entire field-of-view of the patient, making it a suitable option for nonstandard positions in portable radiography. We compare our method with three representative methods in the literature: (1) the beam stopper-based scatter correction method presented by Cai et al.^38^, which shares hardware requirements with our proposal (evaluating only the part of their algorithm devoted to correct projections); (2) the software-based post-processing method proposed by Meng et al.^31^ and that has also been reported to provide a good scatter estimate in planar radiography^27–29^; and (3) the deep learning scatter correction method proposed by Maier et al.^34^. Comparison with other beam stopper methods can be found in the “Supplementary material”.

## Materials and methods

### Proposed method

**Figure 1:**
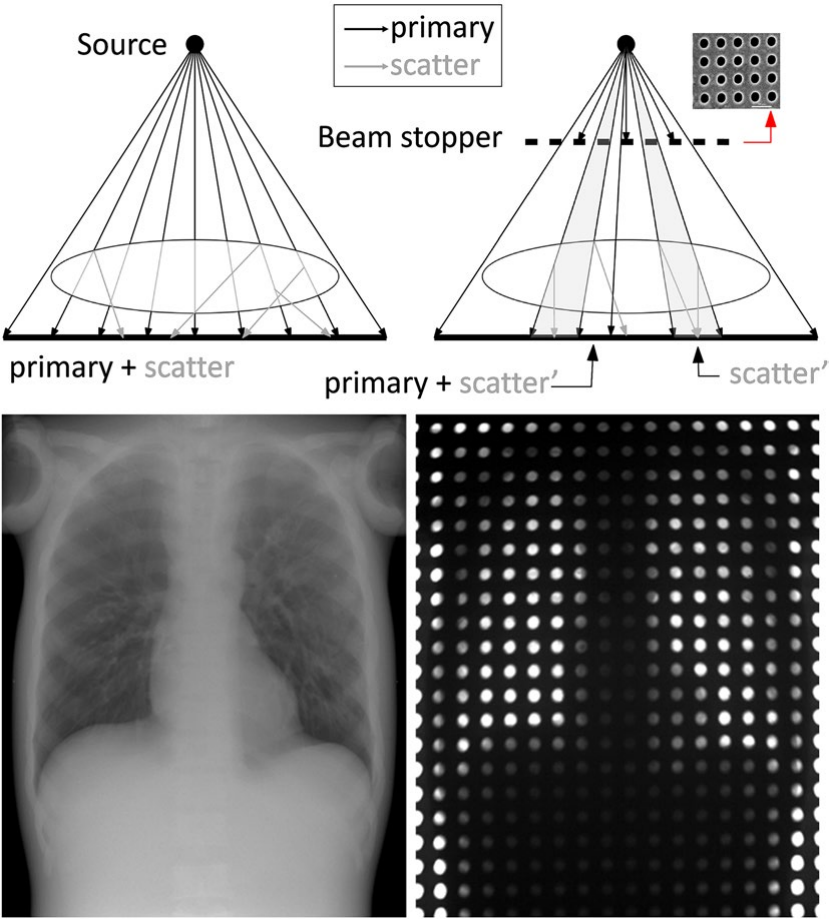
Top: Acquisition geometry for both full-field (left) and partially obstructed projections with the use of the beam stopper (right). Bottom: Example of both projections in a chest phantom acquisition.

The proposed method involves the acquisition of two images: a full-field projection of the patient and a projection partially obstructed with a beam stopper (BS). The beam stopper, which is placed between the source and the patient, comprises a plate of highly absorbent material with a mesh of cylindrical holes. Provided that the beam stopper material guarantees adequate attenuation, the shadow region contains information for the scattered radiation only. Since the beam stopper blocks part of the primary radiation from the source, the shadow region contains a smaller scatter signal (scatter’ in Fig. 1) than the full-field projection obtained without beam stopper.

**Figure 2:**
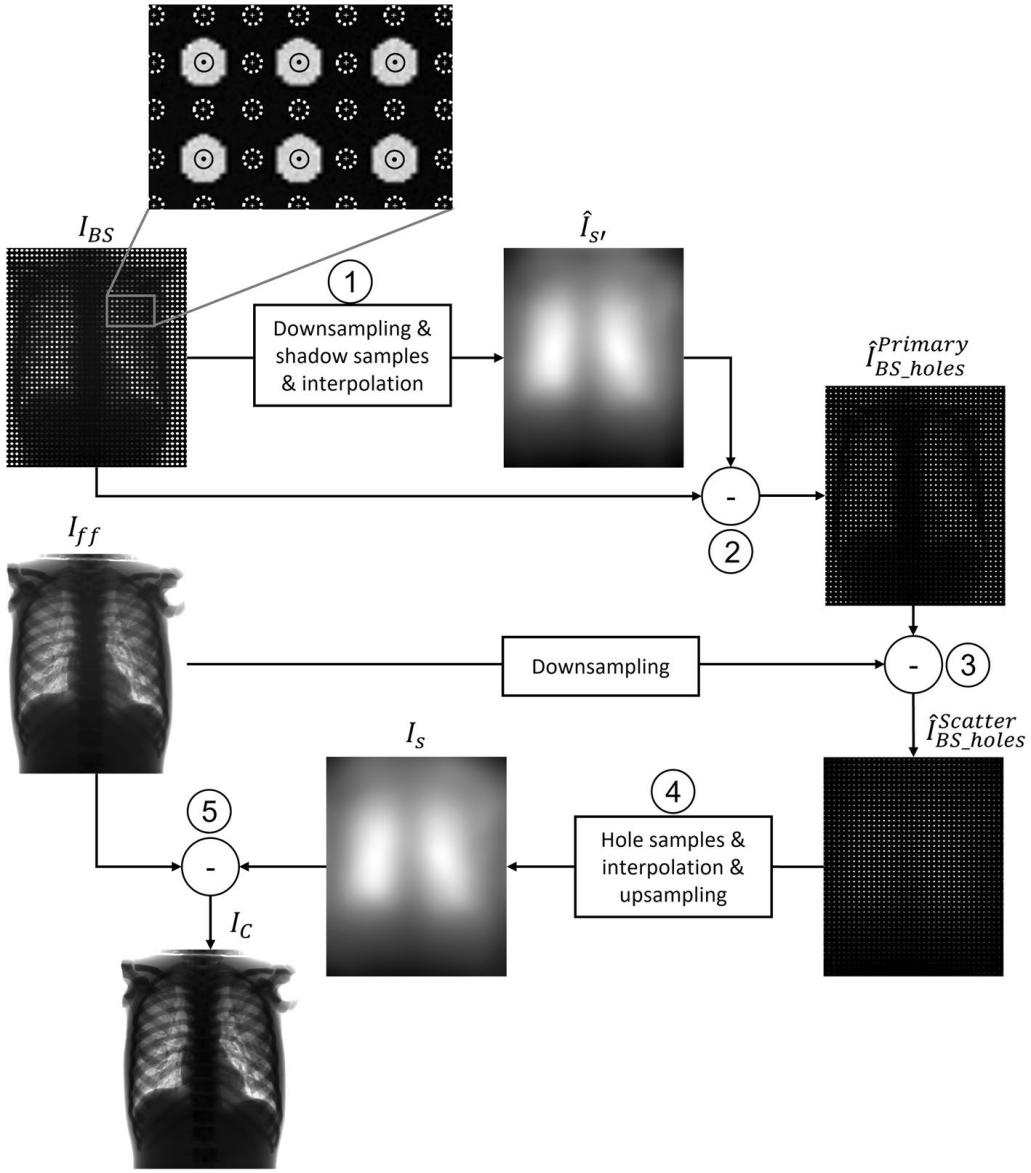
Workflow of the proposed method with a zoom showing the ROIs used for the calculation of the samples of $I_{BS\_holes}$ (black circles) and $I_{BS\_shadow}$ (white dashed circles).

Figure 2 shows the workflow of the proposed scatter correction. The partially obstructed projection $I_{BS}$ is downsampled by a factor of 8 to expedite the 2D fittings with no impact in the final scatter map, given its low-frequency nature, and divided into two regions: (1) the projection of the perforations, $I_{BS\_holes}$, which contains both primary and scattered radiation; and (2) the shadow region, $I_{BS\_shadow}$, which receives only scattered radiation. To separate hole and shadow regions, $I_{BS}$ is binarized by thresholding using the Otsu method^39^. We obtain samples as mean pixel intensity from circles within the projections of the perforations in $I_{BS\_holes}$ (black circles in the zoom shown in Fig. 2) and between them in $I_{BS\_shadow}$ (white dashed circles in the zoom shown in Fig. 2). The radius of the circles is selected to be smaller than the projection of the perforations, thus preventing the penumbra effect at the hole edges.

To interpolate the information missing from the shadow region, we use a locally estimated scatterplot smoothing (LOESS) model^40^, selecting a quadratic function local regression fit with weighted least squares. We used a neighborhood size of 1% of the total amount of samples, value found heuristically as the minimum window to avoid overfitting to noise while maintaining detail. This results in the fitted scatter map $\hat{I}_{{S^{\prime}}}$ (step 1), which represents the reduced scatter signal (scatter’ in Fig. 1). This map is subtracted from a downsampled, partially obstructed projection resulting in the image $\hat{I}_{BS\_holes}^{Primary}$ (step 2). This estimation of the primary signal is then subtracted from the downsampled full-field image $\hat{I}_{ff}$ in order to obtain the final scatter signal in the region of the holes, $\hat{I}_{BS\_holes}^{Scatter}$ (step 3). The missing information is completed by interpolation using the LOESS fitting model, and upsampled to the original image size (step 4). Finally, this estimated scatter map, $I_{s}$, is subtracted from the original full-field image $I_{ff}$ (step 5).

Algorithm 1 shows the pseudocode of the proposed method. The inputs of the algorithm are the full-field projection ($$I_{ff} )$$ and the partially obstructed projection $$\left( {I_{BS} } \right)$$. The output of the algorithm is the scatter-corrected image $$I_{C}$$.
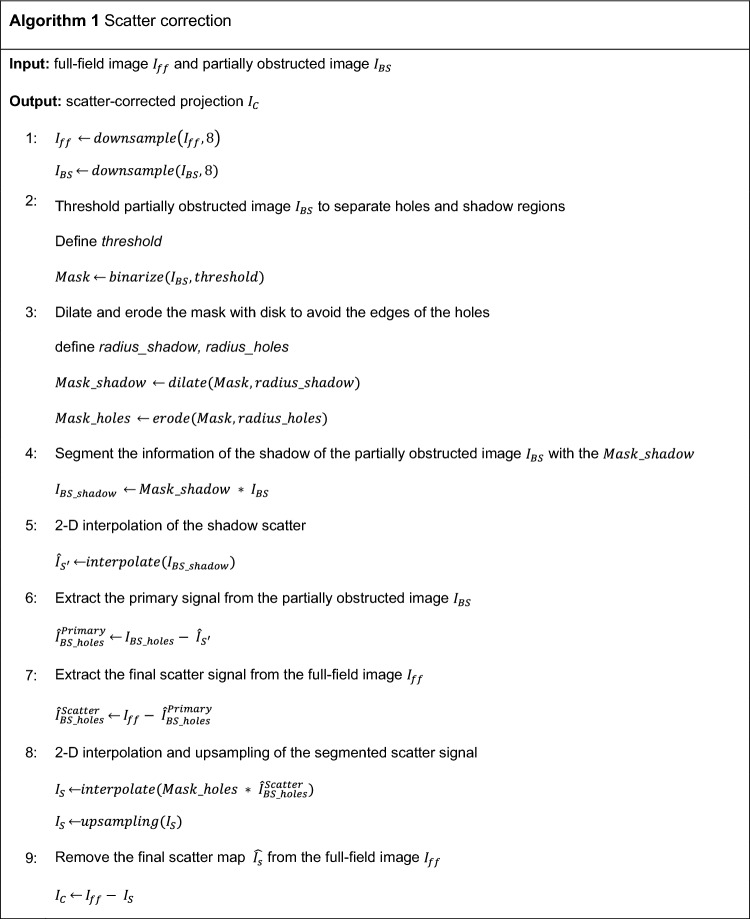


### Evaluation

We evaluated the performance of the proposed method using simulations and real data.

Simulations were generated with the MC-GPU v1.5 simulation package^41,42^, which takes as input the density maps of the different materials in the sample and returns raw images corresponding to the ground truth (Primary), the ideal scatter map (Compton + Rayleigh + Multiscattering), and the full-field image (Primary + Compton + Rayleigh + Multiscattering). The simulated system geometry was based on a real device, the NOVA system (SEDECAL, Madrid, Spain), with a source-to-detector distance of 1800 mm (common in chest X-ray), a distance between the sample and the detector of 50 mm, a detector with a matrix size of 4288 × 3520 pixels (pixel size of 0.1 mm), and a focal spot of 1.2 mm. The two different X-ray spectra, 100 kVp and 120 kVp, were generated using the Spektr toolkit^43^ with a 3-mm Al filter. Although the beam stopper would be close to the X-ray source in a real-world setting, it was placed next to the sample in our simulation in order to speed it up, since computational time depends on the size of the input volume, which includes both the BS and the sample. To maintain the size and separation of the holes in the real-world setting, the BS can easily be translated to the real position by taking into account the magnification. The simulated beam stopper was a 3-mm-thick tungsten plate with holes measuring 5.132 mm in diameter and a distance between centers of 10.264 mm.

Simulations were based on data from 11 real CT volumes: an acquisition of the PBU-60 anthropomorphic phantom (Kyoto Kagaku)^44^ in a Toshiba Aquilion/LB CT scanner, with a matrix size of 349 × 230 × 938 voxels and a voxel size of 0.931 × 0.931 × 0.500 mm, and 10 anonymized clinical CTs from the NSCLC Radiogenomics and MIDRC-RICORD-1B collections of The Cancer Imaging Archive (TCIA)^45^. For the simulation, volumes were segmented into four materials following^46^, with density values of 1.205e10^–3^ g/cm^3^ (air), 1.060 g/cm^3^ (soft tissue), 1.920 g/cm^3^ (bone) and 19.299 g/cm^3^ (tungsten), based on the NIST database^47^.

**Figure 3:**
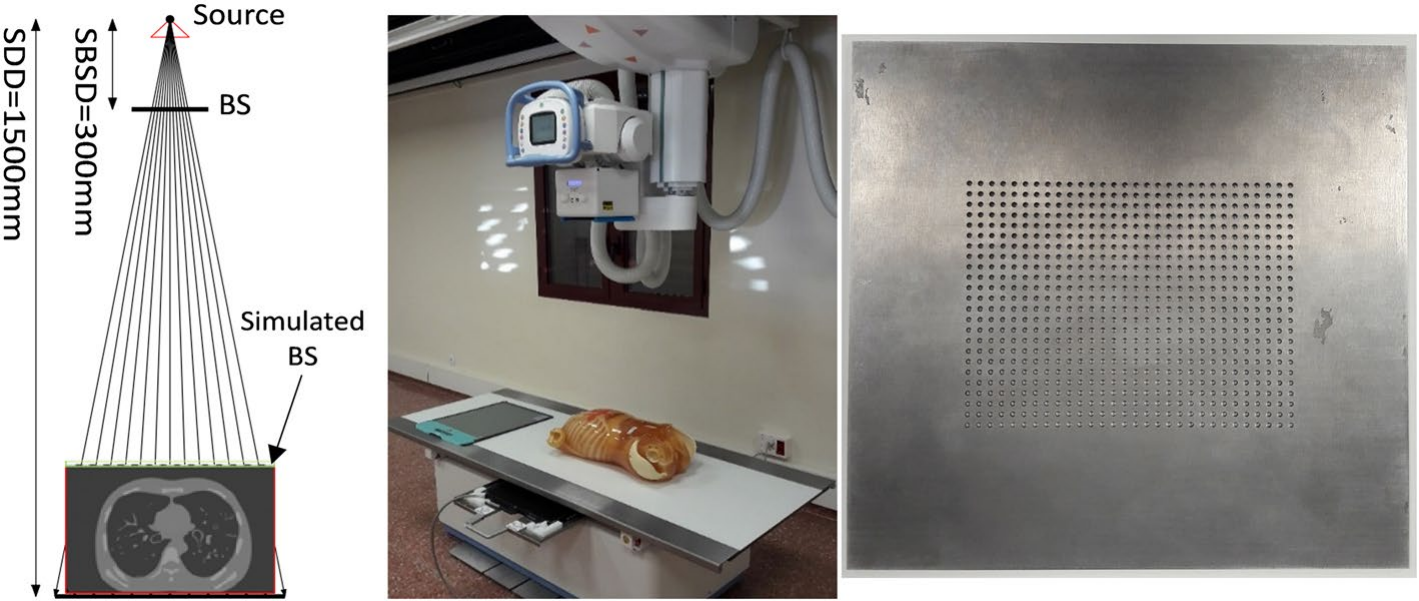
Left: Beam stopper simulation setup. Center: SEDECAL NOVA FA system; on top of the bed, the antiscatter grid and PBU-60 anthropomorphic phantom (Kyoto Kagaku); below the bed, the Perkin Elmer detector. Right: Tungsten beam stopper.

Real acquisitions were carried out with the PBU-60 phantom using a Sedecal NOVA FA system (Fig. 3), with a source-to-detector distance of 1500 mm (limit for bed acquisitions) and a distance between the sample and the detector of 80 mm. The detector was a Perkin Elmer XRpad 4336 with a matrix size of 4288 × 3520 pixels (0.1 mm pixel size). The beam stopper was a tungsten plate (density 19.299 g/cm^3^) with dimensions of 20 cm × 20 cm × 3 mm and a matrix of holes measuring 2 mm in diameter and separated by 4 mm. It was placed at 30 cm from the source, immediately after the collimator, making use of 3D-printed rails for easy access between acquisitions.

We used a tube voltage of 100 kVp, with 3-mm aluminium filtration, selecting a small focal spot (0.6 mm) for the projection partially obstructed by the beam stopper to avoid the partial volume effect on the edges of the holes, and a large focal spot (1.2 mm) for the full-field projection, based on the guidelines for chest radiography. Full-field and partially obstructed projections were obtained with the same mAs value for simplification. The ground truth was approximated by the acquisition of the same sample using an antiscatter grid (aluminium interspacer, 40 l/cm, ratio: 10:1, focal distance: 1500 mm). The SNR was the same as that of the full-field projection ensured by the automatic exposure control of the NOVA FA system using the ionization chamber located in the spine. For comparison, all images were normalized to the same dynamic range, as follows:

$$Img\_Norm = \frac{{Img - min\left( {Img} \right)}}{{\max \left( {Img} \right) - min\left( {Img} \right)}} = \frac{Img - mean(Img < P2\% )}{{mean(Img > P98\% ) - mean(Img < P2\% )}}$$

where P2% and P98% are percentiles 2 and 98, respectively.

We compared our scatter correction method with the following: (1) the post-processing method proposed by Meng et al.^31^, which was based on image dehazing and later used by Kim et al.^27–29^ in radiography; (2) the method based on a beam stopper proposed by Cai et al.^38^; and (3) the deep learning method proposed by Maier et al.^34^, deep scatter estimation (DSE). Below, we provide a summary of the simulation setting and the parameters used in the implementation of these methods.

Meng et al. obtained the corrected or scatter-free image from Eq. (1)

1 $$I_{Meng} \left( x \right) = \frac{I\left( x \right) - A}{{\left[ {max\left( {t\left( x \right),\epsilon } \right)} \right]^{\delta } }} + A,$$

where $I\left( x \right)$ is the input full-field image, $\epsilon$ is a small value to avoid division by zero, $\delta$ an empirical parameter used for fine-tuning the scatter correction of the image, and $t\left( x \right)$ the transmission function that accounts for the primary radiation. We calculated three different values of parameter *A*, each one for a different region of interest (ROI), namely, whole image, lungs, and spine, as the minimum value in the corresponding ROI. The transmission function $t\left( x \right)$ was found by maximizing the contrast resolution using ADMM^32^. Following^31^, values for coefficients C0 and C1 were set as the minimum and maximum values of each ROI, and lambda was set to 1. Finally, δ_global_, δ_lung_, and δ_spine_ were chosen empirically because their results were closer to the ground truth, based on visual inspection, image profiles, and root mean square error (RMSE) (Table 1).

**Table 1:** Parameter values calculated for Meng’s method, optimized for the three regions.

	ϵ	A_global_	A_lung_	A_spine_	δ_global_	δ_lung_	δ_spine_	C_0_	C_1_	$\lambda$
SimPBU100	1e−8	1.5	0.9	2.5	0.3	0.15	0.4	1	4.8	1
SimPat120	1e−8	1.9	1.0	2.8	0.3	0.15	0.4	1	4.8	1
RePBU100	1e−8	1.5	0.9	2.5	0.3	0.15	0.4	1	4.8	1

Consistent with Cai et al.^34^, we simulated a PMMA plate with embedded lead cylinders measuring 3 mm in thickness and 3 mm in diameter and a distance between centers of 11 mm.

The DSE method proposed by Maier et al. was implemented using Pytorch. To train and validate the network, data were generated using the MC-GPU software from 13 real CT volumes (male and female) obtained from the NSCLC Radiogenomics collection TCIA^45^. Using the same acquisition geometry described and a spectrum of 120 kVp, we simulated 533 projections at angular positions ± 20 degrees with 1-degree steps around the antero-posterior radiological position for radiography and 793 projections at angular positions ± 15 degrees with 0.5-degree steps around the antero-posterior radiological position for linear tomosynthesis. Ten CTs (1020 projections) were used to train the network, and three CTs (306 projections) to validate it. The U-Net network was trained with input images of 256×256 (downsampled from projections of 4288﻿×3520 pixels using Nearest-Neighbor interpolation) for 100 epochs with early stopping to choose the model that has the minimum loss over the validation dataset. We used an Adam optimizer with a learning rate of 1.2e−3; as no hints are given in^34^ on how to find the optimum learning rate, this was optimized based on the “learning rate finder” technique proposed by Smith et al. in^48^. Finally, we used a batch size of 30, and defined the loss function as the mean absolute percentage error (MAPE) between the output of the network and the ideal scatter map as in^34^. The achieved minimum validation loss was 4.821%.

Our proposed method and those of Meng et al. and Cai et al. were implemented in MATLAB R2019b on a computer with 32 GB RAM and a CPU Intel Core i7-8700 @ 3.20 GHz. This enabled computational times of around 22 s for the three methods. No efforts were made to optimize the speed.

**Figure 4:**
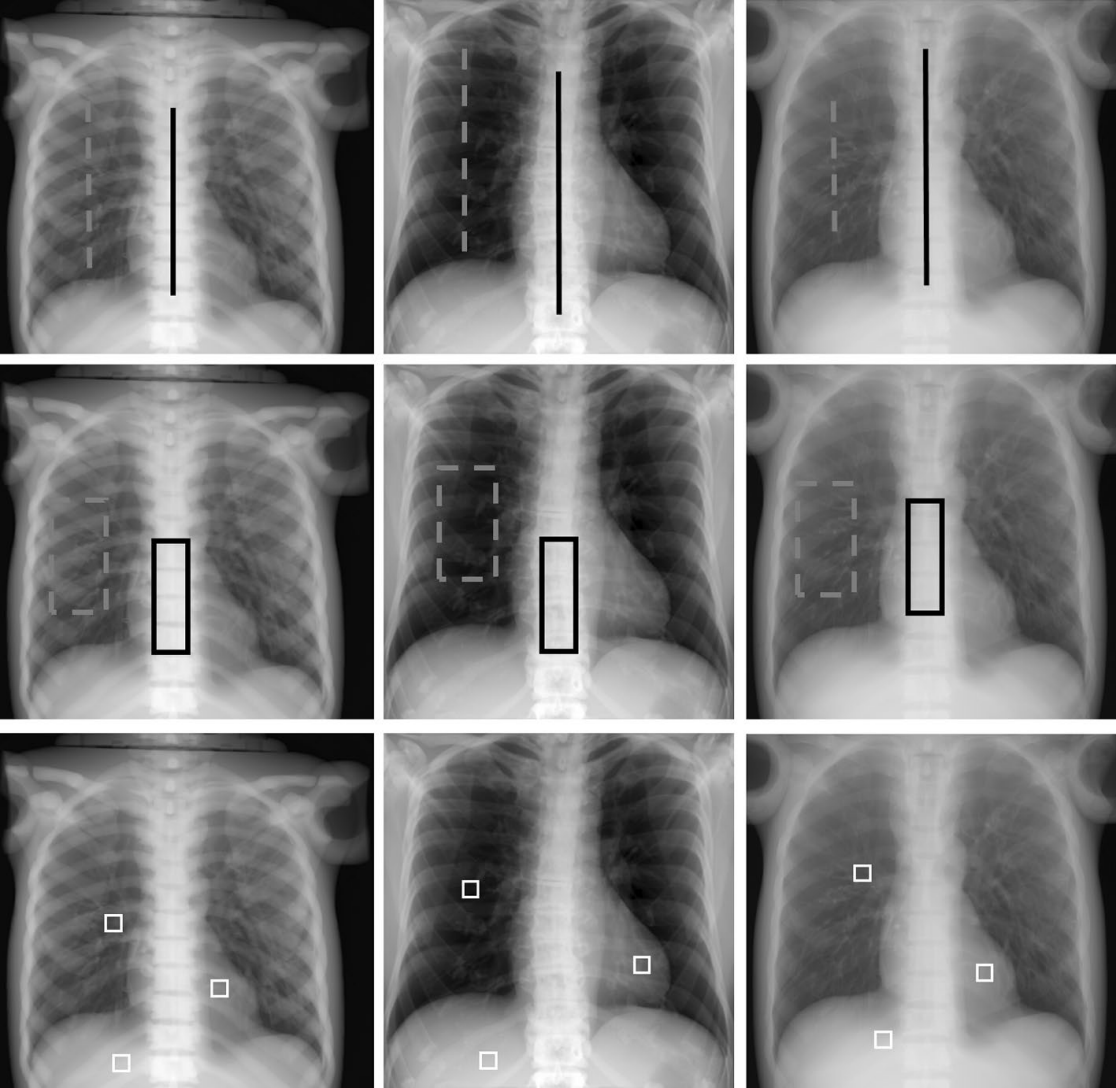
From left to right, projections of the simulated phantom (SimPBU100), one simulated patient (SimPat120) and the real dataset (RePBU100) with image profiles (top) and ROIs for RMSE (middle), SNR and contrast recovery (bottom) calculation. The dashed gray line represents the lung region, and the solid black line represents the spine region.

The evaluation was performed (1) visually, (2) taking profiles from two regions (lung and spine), as shown in top row of Fig. 4, (3) measuring the RMSE between the ground truth and the corrected image in ROIs corresponding to lung (550×1000 pixels) and spine (200×2700) (middle row of Fig. 4), and (4) calculating the following quality measurements:

- Contrast recovery with respect to the ground truth, where the contrast was obtained as the difference between the mean values of 128×128-pixel ROIs placed in the lung and the heart (Fig. 4, bottom).
- SNR measured in an ROI of 128×128 pixels placed in the liver (Fig. 4, bottom).

To assess statistical significance of differences between mean results achieved by the different methods, we used the two-tailed paired Student's T-test, taking p < 0.05 as significance threshold. Statistical significance of differences of variance was evaluated with the F-test. Data normality was assessed by the Kolmogorov–Smirnov test.

Patient dose in the simulated setting was estimated from the percentage of area of highly absorbing material, considering the radiation that traversed the 3 mm of tungsten as negligible. This resulted for the proposed method in a 20% increase when compared with Meng et al. and DSE and half the patient dose when compared with Cai et al.

## Results

**Figure 5:**
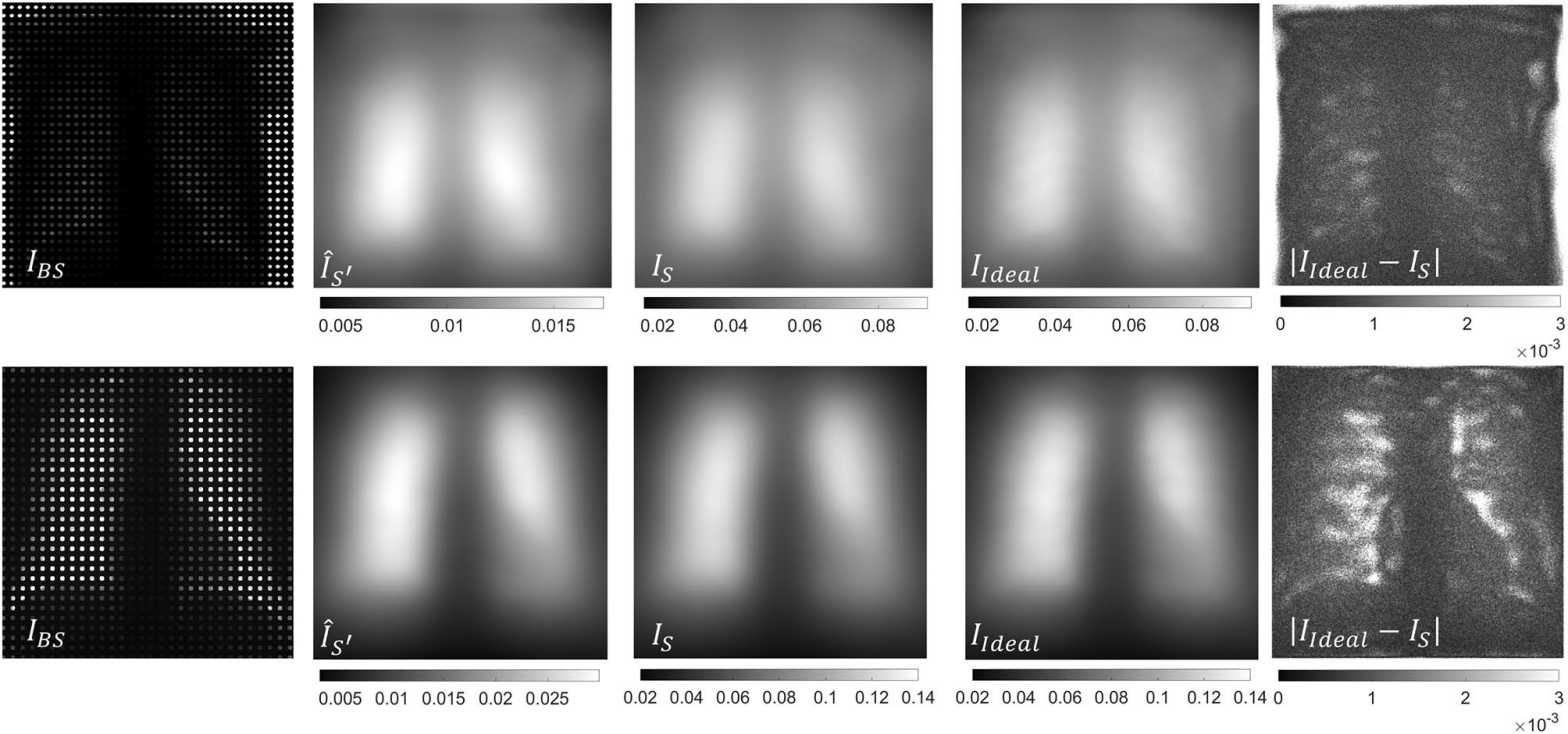
Intermediate images for the simulated phantom SimPBU100 (top) and one of the simulated patients, SimPat120 (bottom). Partially obstructed projection $I_{BS}$, 2D interpolated reduced scatter $\hat{I}_{S^{\prime}}$, corrected scatter estimation $I_{S}$, ideal scatter map, and absolute difference between $I_{Ideal}$ and $I_{S}$.

Figure 5 shows the intermediate steps of the proposed method for the simulated phantom SimPBU100 (top) and one of the simulated patients, SimPat120 (bottom). The values of the interpolated shadow region, $\hat{I}_{{S^{\prime } }}$, are lower than the real scatter because the beam stopper reduces the amount of radiation traversing the patient (scatter’ in Fig. 1). The calculated scatter values corresponding to the full projection are shown in $I_{S}$. We can see that the estimated scatter map correctly recovers the ideal one, except for small details, such as the ribs, which is also visible in the absolute difference image between the ideal and the estimated scatter map. This is due to the downsampling step used to reduce computational cost, which assumes a low frequency for the scatter signal.

**Figure 6:**
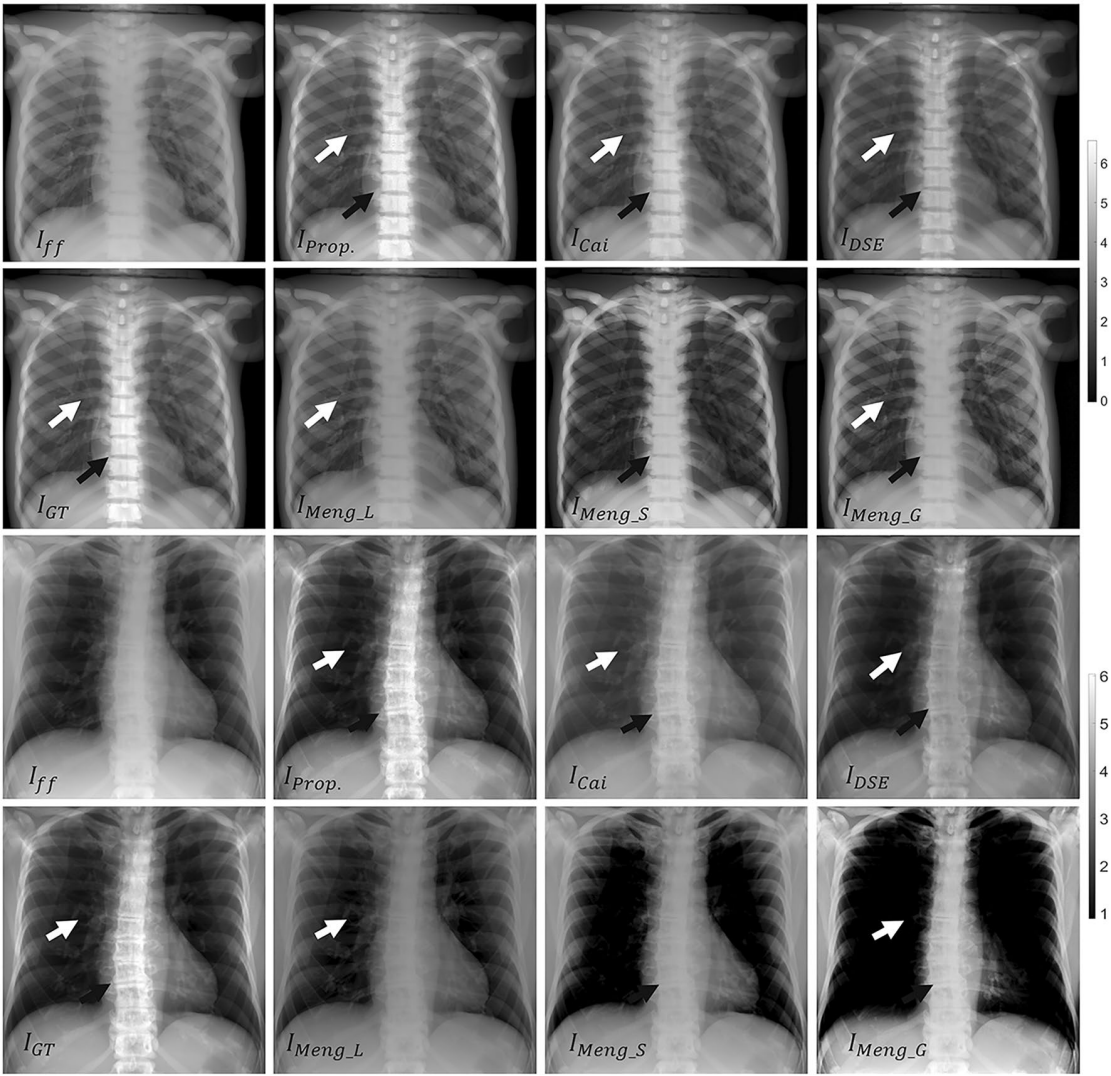
Results for the simulated phantom SimPBU100 (top) and one of the simulated patients, SimPat120 (bottom). Full-field projection ($I_{ff}$), ground truth ($I_{GT}$), and scatter-corrected image with the proposed method ($I_{Prop}$), the method of Cai et al. ($I_{Cai}$), the DSE method ($I_{DSE}$),and the method of Meng et al. for lungs, spine, and global ROIs ($I_{Meng\_L}$, $I_{Meng\_S}$, $I_{Meng\_G}$). White and black arrows highlight noticeable increases in contrast in the lungs and the spine, respectively.

**Figure 7:**
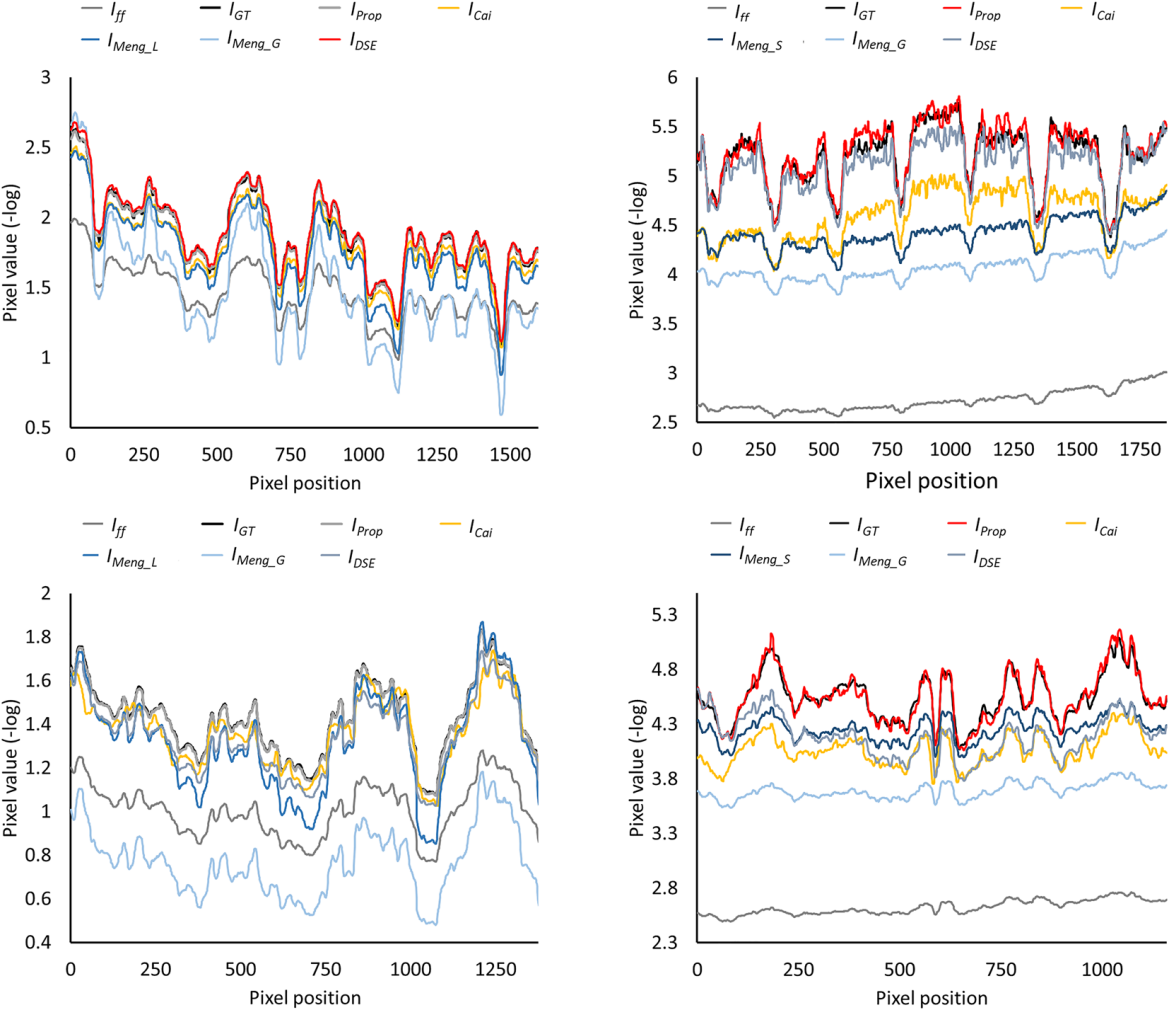
Image profiles for the lungs (left) and the spine (right) shown in Fig. 4 for the simulated phantom SimPBU100 (top) and one of the simulated patients, SimPat120 (bottom). Full-field projection ($I_{ff}$), ground truth ($I_{GT}$), and scatter-corrected image with the proposed method ($I_{Prop}$), the method of Cai et al. ($I_{Cai}$), the DSE method ($I_{DSE}$), and the method of Meng et al. for lungs, spine, and global ROIs ($I_{Meng\_L}$, $I_{Meng\_S}$, $I_{Meng\_G}$).

Figures 6 and 7 show the results obtained with the four methods. The proposed method improves the contrast resolution of the image, independently of the region, with enhanced depiction of the vertebrae and bronchi (white and black arrows in $I_{Prop}$ and $I_{GT}$, Fig. 6). This can be quantitatively assessed in the image profiles, where the proposed method correctly recovers the ground truth values in the area of the spine and lungs (Fig. 7). The correction obtained with A_global_ ($I_{Meng\_G}$) shows limited contrast enhancement in both regions, especially in the spine. Although the approach of Meng et al. increases image contrast in the lungs when using the specific parameter A_lung_ (white arrow in $I_{Meng\_L}$, Fig. 6), there is still some residual scatter signal that hinders the visibility of the bronchi. The use of a spine-specific value, A_spine_ ($I_{Meng\_S}$), does not significantly improve the contrast of the vertebrae in comparison with our method. This can also be objectively observed in the image profiles, where the spine fails to reach the ground truth values (Fig. 7). The method proposed by Cai et al. shows limited overall contrast enhancement compared with our method (white and black arrows, Fig. 6). The difference is especially noticeable in the spine, where the values are farther from the ground truth (Fig. 7). Finally, with the DSE method ($I_{DSE}$ in Fig. 6), contrast enhancement in both regions is also poorer than with the proposed method, showing less conspicuous bronchi (white arrow in $I_{DSE}$, Fig. 6) and vertebrae (Fig. 7).

**Figure 8:**
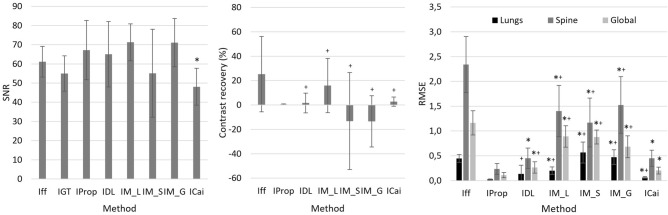
Signal to noise ratio in the liver region, lung contrast recovery and RMSE with respect to the ground truth for the 10 simulated patients. Full-field projection ($I_{ff}$), reference image ($I_{GT}$), and scatter-corrected image with the proposed method ($I_{Prop}$), the DSE method ($I_{DSE}$), and the method of Meng et al. for lungs, spine, and global ROIs ($I_{M\_L}$, $I_{M\_S}$, $I_{M\_G}$). (*) Difference in mean value is statistically significant (p < 0.05) with respect to *I*_*prop*_. (^+^) Difference in standard deviation is statistically significant (p < 0.05) with respect to *I*_*prop*_.

Figure 8 shows the mean value and standard deviation of SNR in liver and the contrast recovery for the 10 simulated patients. SNR was increased by all the methods except for Cai et al. The proposed method showed the best contrast recovery for all patients, while Meng et al. was the method that showed larger disparities between patients.

**Figure 9:**
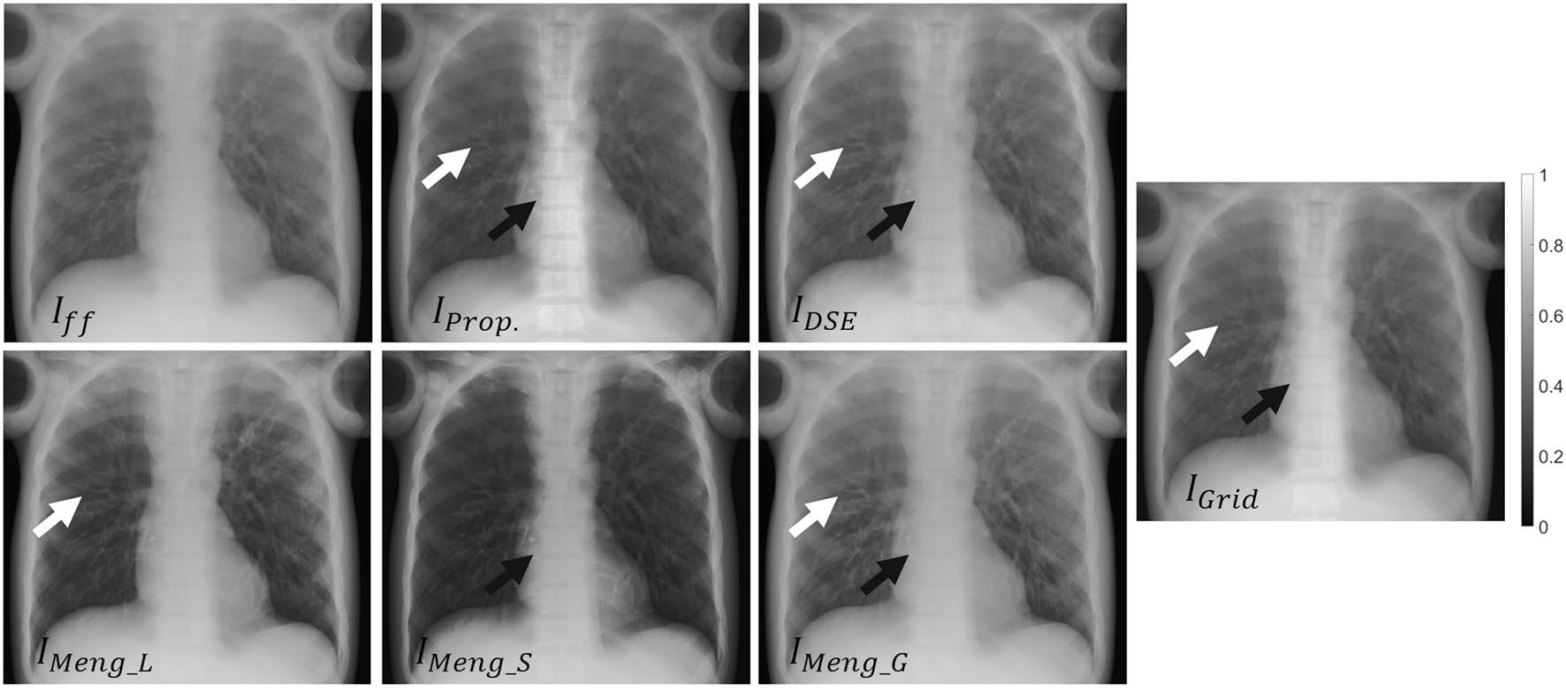
Results for the real dataset RePBU100. Top: Full-field projection ($I_{ff}$) and scatter-corrected images with the proposed method ($I_{Prop}$) and the DSE method ($I_{DSE}$). Bottom: scatter-corrected images with the method of Meng et al. for the lungs, spine, and global ROIs ($I_{Meng\_L}$, $I_{Meng\_S}$, $I_{Meng\_G}$). Middle right: image with antiscatter grid ($I_{Grid}$). White and black arrows highlight noticeable increases in contrast in the lungs and the spine, respectively.

**Figure 10:**
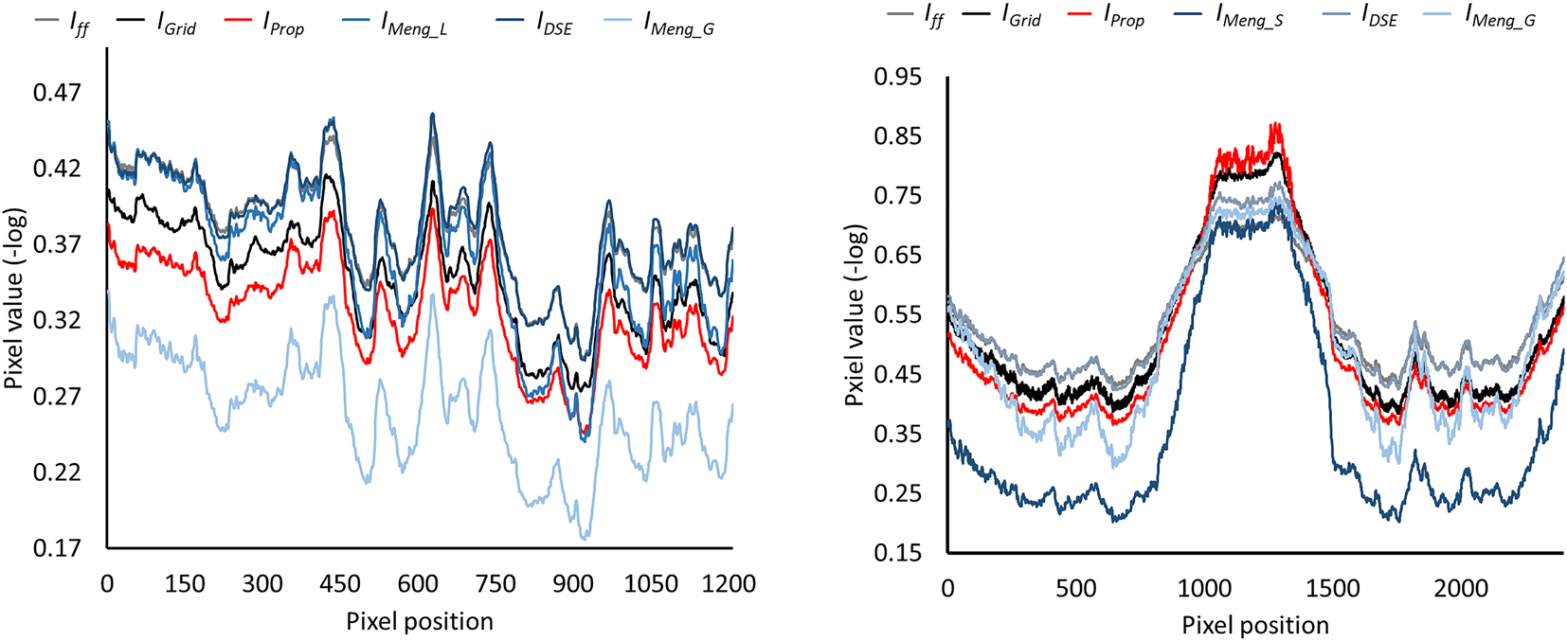
Image profiles for the lungs (left) and the spine (right) shown in Fig. ﻿4 for the real dataset RePBU100. Full-field projection ($I_{ff}$), the image with antiscatter grid ($I_{Grid}$), and scatter-corrected images with the proposed method ($I_{Prop}$), the DSE method ($I_{DSE}$), and the method of Meng et al. for lungs, spine, and global ROIs ($I_{Meng\_L}$, $I_{Meng\_S}$, $I_{Meng\_G}$).

Figures 9 and 10 show the results with the real dataset RePBU100. The method of Meng et al. shows poor contrast enhancement for A_global_ ($I_{Meng\_G}$, Fig. 9), with a slight increase in image contrast, mainly in the lungs for A_lung_ (white arrows in $I_{Meng\_L}$). The DSE method ($I_{DSE}$, Fig. 9) provides some contrast enhancement in the lungs, but the vertebrae located in the lower mediastinal area cannot be distinguished (black arrow in $I_{DSE}$), and spine attenuation values are not properly recovered (Fig. 10, right). The proposed method ($I_{Prop}$) results in an overall improvement in the contrast resolution of the image, which is especially conspicuous in the region of the spine (black arrow in $I_{Prop}$, Fig. 9), and is also able to recover the ground truth values, both along the spine and in the lungs (Fig. 10).

Table 2 shows the RMSE between the scatter-corrected image and the image with the antiscatter grid (approximated ground truth) for all datasets. The proposed method results in the smallest root mean square error in the lungs, spine, and globally.

**Table 2:** RMSE against the ground truth in the lung, spine, and global regions shown in Fig. 5.

Dataset	SimPBU100			Simulated patients (mean ± std)			RePBU100		
	Lungs	Spine	Global	Lungs	Spine	Global	Lungs	Spine	Global
I_Prop_	**0.020**	**0.021**	**0.056**	**0.025 ± 0.006**	**0.234 ± 0.112**	**0.110 ± 0.052**	**0.024**	**0.049**	**0.028**
I_Meng_L_	0.174	1.280	0.555	0.199 ± 0.074*^+^	1.404 ± 0.517*^+^	0.890 ± 0.216*^+^	0.025	0.087	0.053
I_Meng_S_	0.775	0.574	0.616	0.565 ± 0.212*^+^	1.169 ± 0.492*^+^	0.878 ± 0.139*^+^	0.164	0.097	0.131
I_Meng_G_	0.363	0.890	0.419	0.474 ± 0.148*^+^	1.525 ± 0.571*^+^	0.683 ± 0.221*^+^	0.082	0.083	0.060
I_Cai_	0.097	0.558	0.258	0.062 ± 0.018*^+^	0.451 ± 0.162*	0.201 ± 0.070*	–	–	–
I_DSE_	0.022	0.041	0.131	0.136 ± 0.173^+^	0.450 ± 0.206*	0.264 ± 0.116*^+^	0.033	0.066	0.052
I_ff_	0.523	2.109	1.138	0.444 ± 0.080*^+^	2.341 ± 0.564*^+^	1.164 ± 0.244*^+^	0.033	0.101	0.055

(*) Difference in mean value is statistically significant (*p* < 0.05) with respect to *I*_*prop*_.﻿ (^+^) Difference in standard deviation is statistically significant (*p* < 0.05) with respect to *I*_*prop*_*.*

## Discussion and conclusions

We present a method for scatter correction in digital planar chest radiography that provides an image contrast equivalent to that obtained with antiscatter grids while limiting the dose delivered to the patient and removing the geometrical restrictions inherent to the use of antiscatter grids. One of the main advantages of the proposed method is that the correction is based on real patient measurements, thus ensuring that real values are recorded.

For the simulated data, our method showed a better correction than the post-processing method proposed by Meng et al. for all regions—lungs, spine, and global—regardless of the specific selection of heuristic parameter* A* and without the need for parameter tuning. Even though the results of Meng et al. showed increased contrast when the parameter was optimized for a given tissue (lung or spine), ground truth values were not completely recovered, and no optimization was possible for the entire image. This is possibly because, contrary to the assumption of homogeneity of the haze in this method, scatter differs with the density of the material traversed. Although scatter correction based on Cai et al. was good in the lung region, our method showed better overall contrast enhancement, consistent along all the samples. The proposed method showed the best contrast recovery for all patients, while Meng et al. was the method that showed larger disparities between patients, highly depending on the original contrast of the image (the processed images of the other 9 patients can be seen in the “Supplementary material”). Finally, the DL method from Maier et al. provided a good estimation of the lung region, where most of the scatter is concentrated, although the depiction of the vertebrae remained blurrier in the region of the spine. Despite the clear advantage of the proposed method, some of the differences observed did not reach statistical significance. This is surely due to the low sample size (N = 10) which does not allow for a high statistical power in detecting such differences. The reduced sample size also justifies why we did not attempt to correct p-value for multiple comparisons, which is a limitation of the statistical approach.

Meng et al. reported similar performance in real data to that observed in simulations, while DSE performed worse with real data; this was expected, since the network was trained only with simulated data. Differences with the real data may arise owing to simplifications during simulations, such as the X-ray spectrum, the response of the detector, and the material segmentation for the creation of the voxelized volumes. We cannot rule out the possibility that a deep learning approach based on real patient datasets with and without the grid could outperform our approach. Nevertheless, this would require a large clinical trial and may pose external validity issues, only if the device or its configuration is changed.

The proposed method showed the best results with real data, achieving higher contrast resolution than the image obtained with the antiscatter grid, especially in the spine, which could be seen as a slight overcorrection in the values obtained, possibly because we used a one dimensional antiscatter grid of 40 lines per cm which still allows scatter radiation to reach the detector. Therefore, the “ground truth” image is suboptimal. We also note that the unusual contrast observed in the spine compared with clinical radiographs might be due to the fact that the PBU-60 phantom is constructed with solid vertebrae. Further evaluation of the proposed method in real patients is warranted to verify its applicability.

The current beam stopper design was effective, although further improvements could be made. Future investigations might try aligning the holes onto the X-ray beam to minimize partial volume, optimizing the thickness of the plate to eliminate radiation in the shadow, or optimizing diameter and separation of the holes to reduce the radiation dose in the second acquisition by limiting the exposed areas: a smaller hole size will result in a lower extra dose delivered to the patient, but also in poorer scatter sampling.

In order to implement this method in a clinical setting, the beam stopper could be placed next to the X-ray tube, considerably reducing its size and cost. Such an approach will require an automated mechanism for fast insertion and removal to avoid patient artifacts between the two acquisitions. Nevertheless, since the second beam stopper acquisition is only used to create the scatter map, the low-frequency nature of this map means that we do not expect much effect from small patient movements. Future evaluations based on real data should include optimization of acquisition parameters for the partially obstructed projection to further decrease the dose.

In summary, we present a new method for scatter correction in digital chest radiography based on direct patient measurement, thus avoiding the biases of post-processing methods, with results similar to those recorded with an antiscatter grid but with a highly reduced radiation dose. The method can be used in nonconventional imaging techniques, such as portable radiography, where training datasets needed for deep-learning approaches would be very difficult to obtain.

## Supplementary Information

Supplementary Information.

## Data Availability

All relevant data are available from the Zenodo database, under https://doi.org/10.5281/zenodo.6821727.
