# Supplementary material for: A novel beam stopper-based approach for scatter correction in digital planar radiography
Source: Sci Rep. 2023 May 31;13:8795. doi: 10.1038/s41598-023-32764-5 (PMC10232419; doi:10.1038/s41598-023-32764-5)
Supplement: Supplementary file 1 — Supplementary Information. [file 41598_2023_32764_MOESM1_ESM.pdf]

Supplementary information for "A novel beam stopper–based approach for scatter correction in digital planar radiography"

Here we add in the comparison the results of a simplified method based on a global factor instead of the local factor used in the proposed method, which is calculated from the ratio of the two projections in the regions of the holes (steps common to the proposed method are shown in gray):

**Simplified algorithm**
**Input:** full-field image I_{ff} and partially obstructed image I_{BS}<br>**Output:** scatter-corrected projection I_C<br>1: $I_{ff} \leftarrow downsample(I_{ff}, 8)$<br>$I_{BS} \leftarrow downsample(I_{BS}, 8)$<br>2: Threshold partially obstructed image I_{BS} to separate holes and shadow regions<br>Define *threshold*<br>$Mask \leftarrow binarize(I_{BS}, threshold)$<br>3: Dilate and erode the mask with disk to avoid the edges of the holes<br>define *radius_shadow, radius_holes*<br>$Mask\_shadow \leftarrow dilate(Mask, radius\_shadow)$<br>$Mask\_holes \leftarrow erode(Mask, radius\_holes)$<br>4: Segment the information of the shadow of the partially obstructed image I_{BS} with the $Mask\_shadow$<br>$I_{BS\_shadow} \leftarrow Mask\_shadow * I_{BS}$<br>5: 2-D interpolation of the shadow scatter<br>$\hat{I}_{S'} \leftarrow interpolate(I_{BS\_shadow})$<br>6: Ratio in the holes<br>$R \leftarrow I_{ff}/I_{BS}$<br>$R_{holes} \leftarrow Mask\_holes * R$<br>7: Calculate global factor GF<br>$GF \leftarrow mean(R_{holes})$<br>9: Remove the final scatter map $\widehat{I_s}$ from the full-field image I_{ff}<br>$I_C \leftarrow I_{ff} - GF * upsampling(\hat{I}_{S'})$

The calculated factors were 2.7, 2.5 and 2.65 for SimPBU100, SimPat120: 2.5 and RePBU100, respectively.

Evaluation on simulated data showed that the global factor (I_{GF} in Figure S1) shows limited contrast improvement in the lungs (white arrows in I_{GF}, Figure S1) and especially in the spine, with the vertebrae separation is still hinder by scatter (black arrows in I_{GF}, Figure S1). This is also appreciated in Figure S2, where the values do not reach the ground truth.

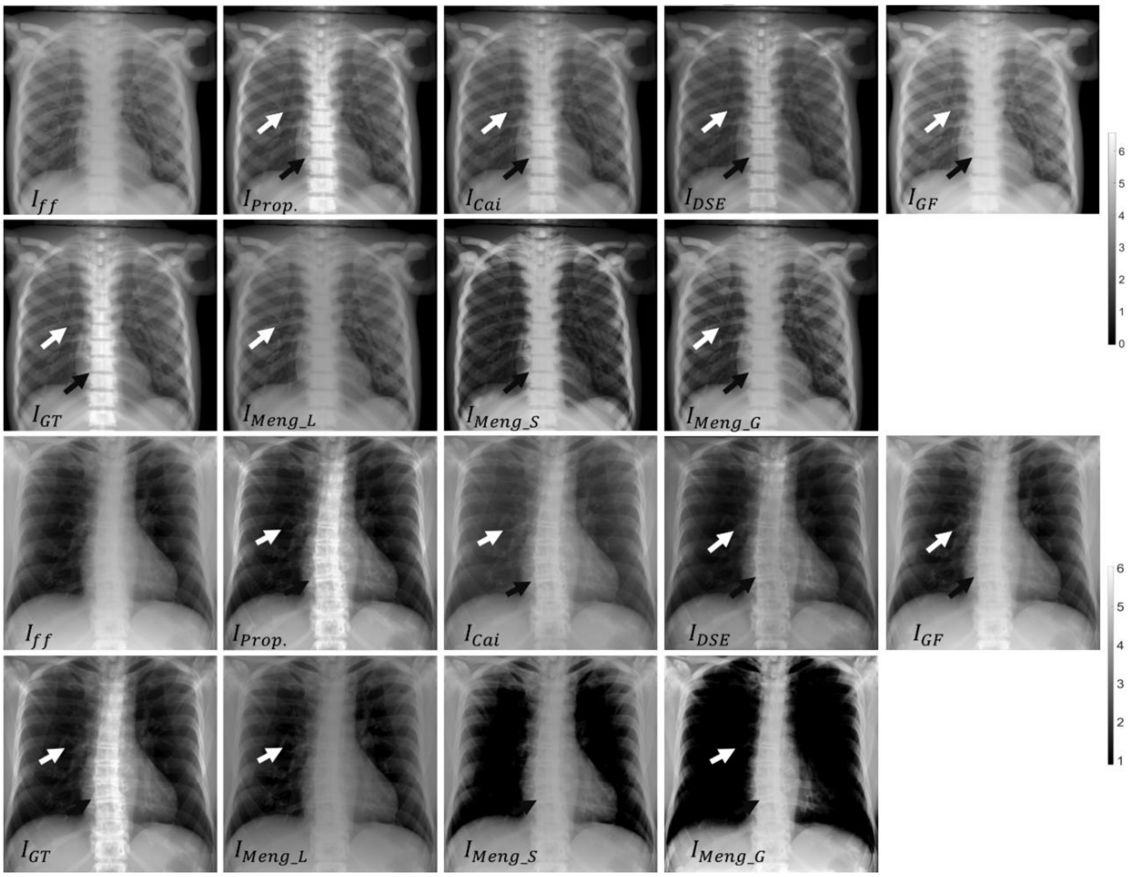

Figure S1. Results for SimPBU100 (top) and SimPat120 (bottom). Full-field projection (I_{ff}), ground truth (I_{GT}), and scatter-corrected image with the proposed method (I_{Prop}), the method of Cai et al. (I_{Cai}), the DSE method (I_{DSE}), the global factor (I_{GF}), and the method of Meng et al. for lungs, spine, and global ROIs (I_{Meng_L}, I_{Meng_S}, I_{Meng_G}). White and black arrows highlight noticeable increases in contrast in the lungs and the spine, respectively.

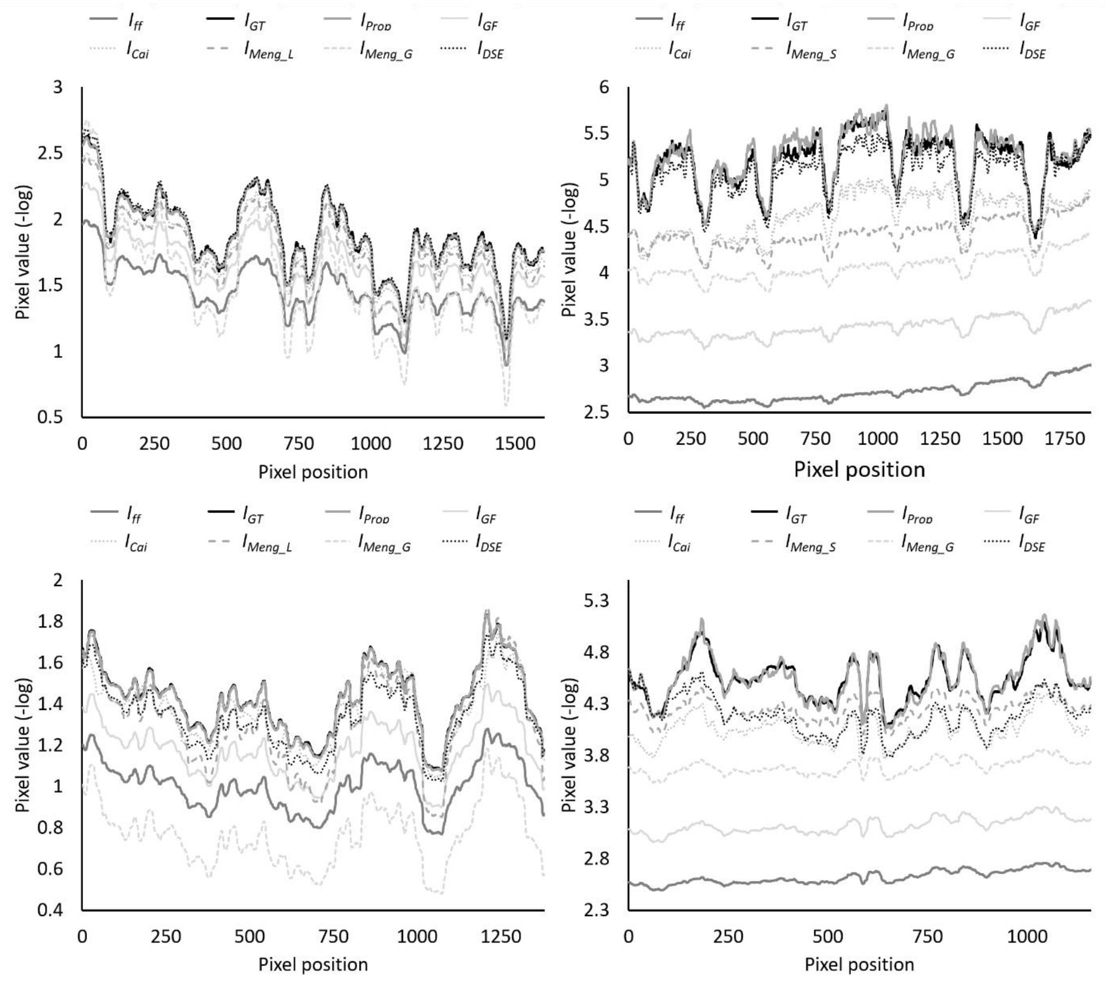

Figure S2. Image profiles for the lungs (left) and the spine (right) shown in Figure 5 for the simulated datasets SimPBU100 (top) and SimPat120 (bottom). Full-field projection (I_{ff}), ground truth (I_{GT}), and scatter-corrected image with the proposed method (I_{Prop}), the method of Cai et al. (I_{Cai}), the DSE method (I_{DSE}), the global factor (I_{GF}), and the method of Meng et al. for lungs, spine, and global ROIs (I_{Meng_L}, I_{Meng_S}, I_{Meng_G}).

In real data, the correction with a global factor (I_{GF}) showed poorer contrast enhancement than the proposed method in both regions (black and white arrows in I_{GF} in Figure S3), and is also unable to recover the ground truth values in both regions of spine and lungs (Figure S4).

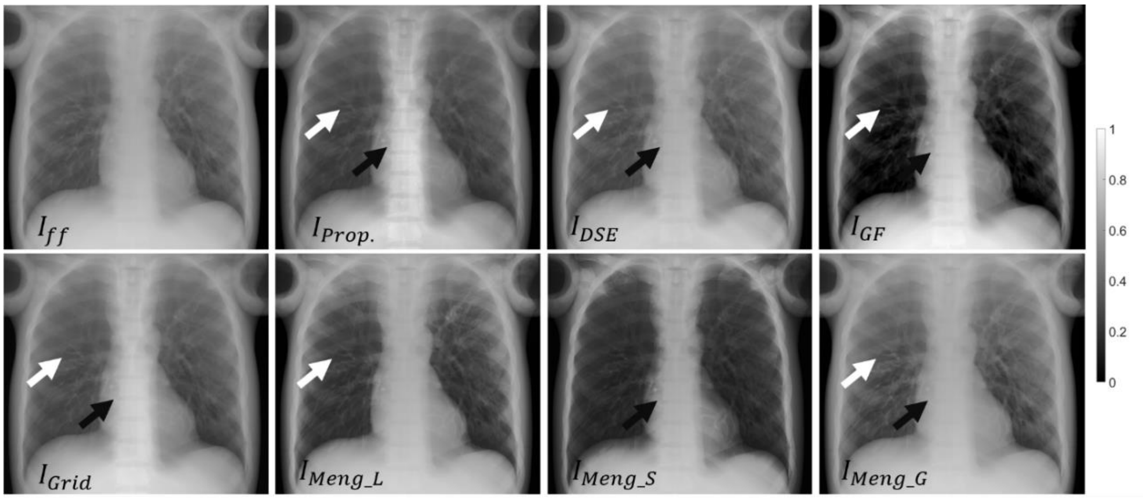

Figure S3. Results for the real dataset RePBU100. Top: Full-field projection (I_{ff}) and scatter-corrected images with the proposed method (I_{Prop}), the DSE method (I_{DSE}) and the global factor (I_{GF}). Bottom: image with antiscatter grid (I_{Grid}), and scatter-corrected images with the method of Meng et al. for the lungs, spine, and global (I_{Meng_L}, I_{Meng_S}, I_{Meng_G}). White and black arrows highlight noticeable increases in contrast in the lungs and the spine, respectively.

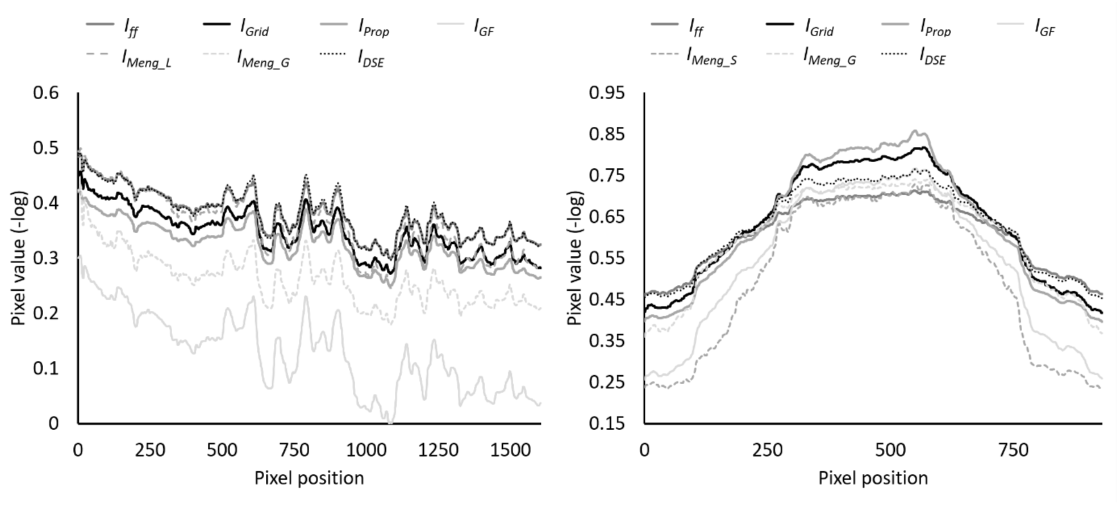

Figure S4. Image profiles along the lungs (left) and along the spine (right) shown in Figure 5 for the real dataset RePBU100. Full-field projection (I_{ff}), the image with antiscatter grid (I_{Grid}), and scatter-corrected image with the proposed method (I_{Prop}), the DSE method (I_{DSE}), the global factor (I_{GF}) and the method of Meng et al. for lungs, spine, and global ROIs (I_{Meng_L}, I_{Meng_S}, I_{Meng_G}).

Table S1 shows the RMSE between the scatter-corrected image and the image with the antiscatter grid (approximated ground truth) for the three datasets. The proposed method results in the smallest root mean square error in the lungs, spine, and globally.

Dataset	SimPBU100			SimPat120			RePBU100		
	**Lungs**	**Spine**	**Global**	**Lungs**	**Spine**	**Global**	**Lungs**	**Spine**	**Global**
I_{Prop}	**0.0197**	**0.0212**	**0.0566**	**0.0148**	**0.1036**	**0.0489**	**0.0245**	**0.0494**	**0.0280**
I_{GF}	0.1435	0.3820	0.5768	0.1510	0.5221	0.7023	0.0931	0.0743	0.1544
I_{Meng_L}	0.1739	1.2804	0.5547	0.0958	1.2041	0.5270	0.0253	0.0875	0.0528
I_{Meng_S}	0.7755	0.5739	0.6165	0.9805	0.3516	0.7948	0.1637	0.0969	0.1311
I_{Meng_G}	0.3629	0.8900	0.4192	0.6331	0.8845	0.4755	0.0822	0.0833	0.0597
I_{Cai}	0.0970	0.5581	0.2585	0.0908	0.5790	0.2984	-	-	-
I_{DSE}	0.0222	0.0414	0.1307	0.0569	0.1427	0.2390	0.0331	0.0662	0.0524
I_{ff}	0.5234	2.1092	1.1382	0.4573	1.9267	1.0246	0.0332	0.1012	0.0551

Table S1. RMSE against the ground truth in the lung, spine, and global regions shown in Figure 4.
